# Supplementary material for: Serum Epiplakin Might Be a Potential Serodiagnostic Biomarker for Bladder Cancer
Source: Cancers (Basel). 2021 Oct 14;13(20):5150. doi: 10.3390/cancers13205150 (PMC8534213; doi:10.3390/cancers13205150)
Supplement: Supplementary file 1 [file cancers-13-05150-s001.zip › cancers-1388846-supplementary.pdf]

Table S1. Univariate and multivariate Cox proportional hazards regression analyses using serum samples to predict NMIBC recurrence.

	Univariate			Mulitivariate		
Factor	HR	95%CI	p	HR	95%CI	p
Serum epiplakin ≧873 (reference: <873)	0.30	0.74-1.23	0.09	0.19	0.02-1.41	0.10
Grade 3 (reference: G1/G2)	1.16	0.36-3.70	0.80	3.10	0.35-27.2	0.31
Urine cytology (reference: <Ⅲb)	0.98	0.28-3.36	0.97	0.45	0.03-5.86	0.54
pT1 (reference: <pT1)	0.66	0.20-2.21	0.50	0.49	0.31-7.60	0.61

HR: hazard ratio, CI: confidence interval.

Table S2. Clinical and pathological characteristics of patients with low and high epiplakin expressions according to immunohistochemistry.

Characteristics	Epiplakin		p-value
	Low (n=80)	High (n=47)	
Age (years) (%)			0.28
≦65	38 (47.5)	27 (57.4)	
>65	42 (57.5)	20 (42.6)	
Sex (%)			0.23
Male	61 (76.3)	40 (85.1)	
Female	19 (23.8)	7 (14.9)	
T stage (%)			0.15
pTa	0	2 (4.3)	
pTis	1 (1.3)	3 (6.4)	
pT1	14 (17.5)	8 (17.0)	
pT2	20 (25.0)	12 (25.5)	
pT3	32 (40.0)	12 (25.5)	
pT4	13 (16.3)	10 (21.3)	
N stage (%)			0.56
pN0	54 (67.5)	34 (72.3)	
≧pN1	21 (26.3)	12 (25.5)	
Unknown	5 (6.3)	1 (2.1)	
Grade (%)			0.18
G1/2	33 (41.3)	19 (40.4)	
G3	47 (58.8)	26 (55.3)	
Unknown	0	2 (4.3)	
LVI (%)			0.21
Negative	24 (30.0)	16 (34.0)	
Positive	44 (55.0)	19 (40.4)	
Unknown	12 (15.0)	12 (25.5)	
CIS (%)			0.21
Negative	66 (82.5)	42 (89.4)	
Positive	14 (17.5)	4 (8.5)	
Unknown	0	1 (2.1)	
Recurrence (%)			0.92
Yes	35 (43.8)	21 (44.7)	
No	45 (56.3)	26 (55.3)	
Cancer death (%)			0.88
Yes	30 (37.5)	17 (36.2)	
No	50 (62.5)	30 (63.8)	
Follow-up, month (IQR)	114.78 (2.67-731)	70.8 (1.8-663.8)	

Unless otherwise stated, values are medians with ranges in parentheses or numbers of patients with percentages in parentheses.

LVI: lymphovascular invasion, CIS: carcinoma in situ, IQR: interquartile range

Table S3. Univariate and multivariate Cox proportional hazards regression analyses using immunohistochemical staining to predict recurrence.

	Univariate			Multivariate		
Factor	HR	95%CI	p	HR	95%CI	p
Epiplakin score: high (reference: low)	0.75	0.44-1.27	0.28	0.72	0.39-1.33	0.29
pT2-4 (reference: <pT2)	2.24	1.14-4.41	0.02	1.78	0.68-4.67	0.24
pN positive (reference: pTN negative)	2.86	1.67-4.89	<0.01	2.04	1.09-3.81	0.03
Grade 3 (reference: G1/G2)	1.33	0.80-2.22	0.27	1.02	0.54-1.92	0.95
LVI positive (reference: negative)	2.52	1.35-4.71	<0.01	1.73	0.85-3.53	0.13
CIS positive (reference: negative)	0.64	0.29-1.41	0.27	0.93	0.39-2.24	0.88

HR: hazard ratio, CI: confidence interval, LVI: lymphovascular invasion, CIS: carcioma in situ.

Table S4. Univariate and multivariate Cox proportional hazards regression analyses using immunohistochemical staining to predict cancer-specific survival.

	Univariate			Multivariate		
Factor	HR	95%CI	p	HR	95%CI	p
Epiplakin score: high (reference: low)	0.64	0.36-1.16	0.14	0.59	0.29-1.19	0.14
pT2-4 (reference: <pT2)	2.01	0.98-4.13	0.06	1.62	0.56-4.68	0.37
pN positive (reference: pN negative)	3.03	1.69-5.45	<0.01	2.38	1.20-4.71	0.01
Grade 3 (reference: G1/G2)	1.46	0.83-2.56	0.20	1.14	0.58-2.26	0.7
LVI positive (reference: negative)	2.43	1.23-4.80	0.01	1.46	0.66-3.21	0.35
CIS positive (reference: negative)	0.42	0.15-1.18	0.10	0.47	0.14-1.56	0.22

HR: hazard ratio, CI: confidence interval, LVI: lymphovascular invasion, CIS: carcioma in situ.

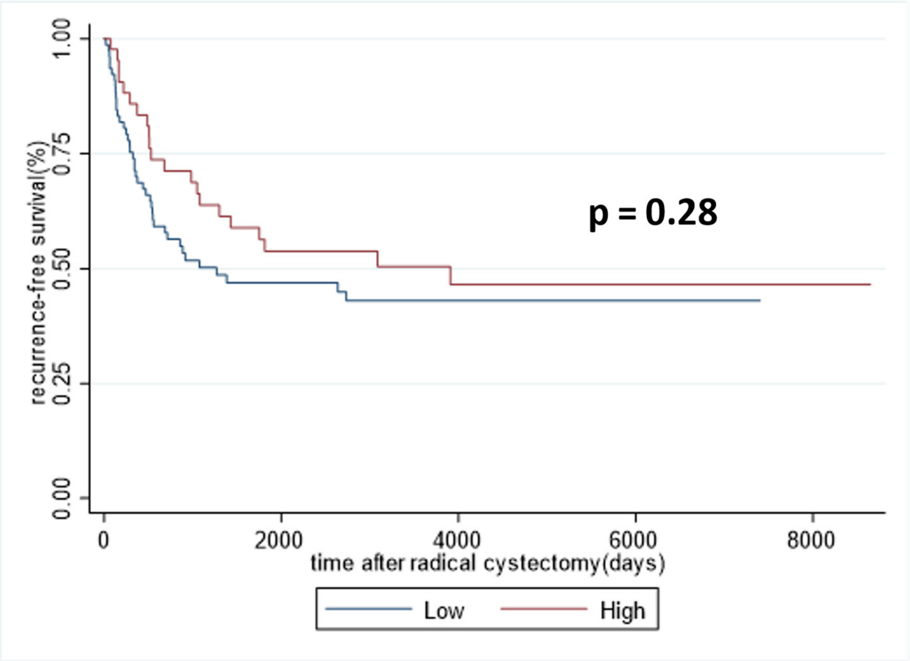

Figure S1. Survival analysis using a Kaplan-Meier curve to determine recurrence-free survival for high and low epiplakin expression.

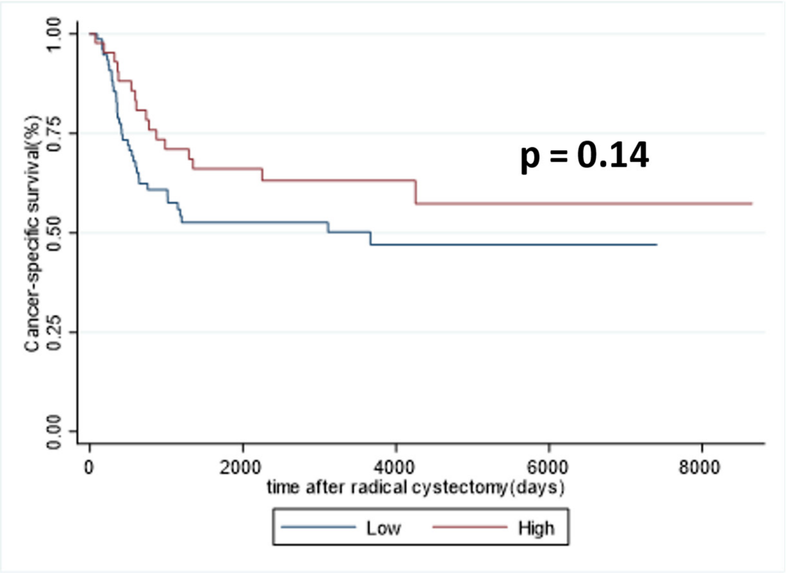

Figure S2. Survival analysis using a Kaplan-Meier curve to determine cancer-specific survival for high and low epiplakin expression.
